# The prevalence and correlates of self-reported hearing impairment in the Ibadan Study of Ageing

**DOI:** 10.1016/j.trstmh.2010.03.009

**Published:** 2010-08

**Authors:** Akeem O. Lasisi, Taiwo Abiona, Oye Gureje

**Affiliations:** a Department of Otorhinolaryngology, College of Medicine, University of Ibadan, Ibadan, Oyo State, Nigeria; b Department of Psychiatry, College of Medicine, University of Ibadan, Ibadan, Oyo State, Nigeria

**Keywords:** Hearing impairment, Elderly, Prevalence, Demographic factors, Lifestyle, Nigeria

## Abstract

This cohort study of 1302 persons aged ≥65 years, conducted in the Yoruba-speaking regions of Nigeria, determines the prevalence and correlates of hearing impairment (HI) in the elderly population. Self-reports of HI and its putative risk factors among several indices were obtained using face-to-face interviews, and confirmed by observer's evaluation. Hearing impairment was found in 79 respondents, giving a prevalence of 6.1%. Gender difference was not significant but increasing age was associated with higher prevalence. Logistic regression analysis, adjusted for age and sex, revealed that history of recurrent suppurative otitis media [odds ratio (OR) = 4.6, 95% CI 2.34–8.99, *P* = 0.01], head injury (OR = 2.2, 95% CI 1.14–4.26, *P* = 0.02) and current hypertension (OR = 2.1, 95% CI 1.18–3.57, *P* = 0.01) were significantly associated with HI. No identifiable risk factors were found in 32 (40.5%) of the 79 respondents with HI. We conclude that the prevalence of HI among the elderly in Nigeria is comparable to reports from other countries. Identified risk factors were preventable or controllable. The large proportion of elderly with no identifiable risk factors, presumably presbyacusis, suggests a need for further study. The strategies for control of these risk factors and hearing aid support should be integrated into health care policy initiatives for elderly persons in sub-Saharan Africa.

## 1 Introduction

Hearing impairment (HI) is the most common sensory deficit among older adults and its effects can be socially and psychologically devastating, leading to loneliness, isolation, anxiety and depression, and associated with other sensory impairment.^1^, ^2^ The projected global rise in the proportion of persons aged ≥65 years is likely to be associated with increasing prevalence of HI among the elderly.^3^, ^4^

The control of risk factors offers the prospect of stemming the rise in the prevalence of HI. Studies from developed countries have documented age, noise, head trauma and chronic medical illnesses as significant risk factors for HI.^3^, ^5^, ^6^ Risk factors may be different in developing countries. For example, in view of large sections of the population residing in rural areas in developing countries, noise may be a less important factor. On the other hand, poor access to medical services may mean that medical conditions that could otherwise be promptly treated may become chronic and therefore predispose to HI. For example, poorly controlled hypertension or diabetes may predispose to HI through the occurrence of chronic arteriosclerosis which in turn causes a reduction in the blood supply to the inner ear.^4^, ^5^, ^6^ It is also plausible to speculate that the presence of chronic recurrent rhinosinusitis and chronic ear discharge will predispose to HI in the elderly.

Even though the majority of elderly persons in the world reside in developing countries and the proportion of the elderly population in these developing countries is projected to rise even further, there has been little study of the major causes of disability among them. Specifically, there is a paucity of studies addressing the prevalence and correlates of HI in the elderly in these countries with a consequent gap in our knowledge about effective strategies to prevent the problem.^5^, ^6^ In this report, we present the results of an epidemiologic study of hearing loss in a community sample of elderly persons. The report examines the prevalence and putative risk factors associated with hearing loss in the elderly.

## 2 Methods

### 2.1 Sampling

The Ibadan Study of Ageing is a longitudinal cohort study of the mental and physical health status as well as the functioning and disability of elderly persons (aged ≥65 years) residing in the Yoruba-speaking areas of Nigeria, which consists of eight contiguous states in the southwestern and northcentral regions (Lagos, Ogun, Osun, Oyo, Ondo, Ekiti, Kogi and Kwara). The population of these states is approximately 25 million people, which is about 22% of the Nigerian population. The baseline survey was conducted between November 2003 and August 2004 and the methodology has been described in full elsewhere;^7^, ^8^ only a brief summary is provided here. Respondents were selected using a multistage stratified area probability sampling of households. In households with more than one eligible person (aged ≥65 years and fluent in Yoruba, the language of the study), the Kish table selection method was used to select one respondent.

### 2.2 Data collection

Face-to face interviews were carried out at baseline in 2003 on 2152 respondents who provided consent to participate, representing a response rate of 74.2%.

An annual three-wave follow-up of the cohort was begun in 2007. Of the baseline sample, 1413 were alive in 2007. This cohort was enlarged by the addition of 461 new respondents, thus resulting in a total of 1874. A second-wave assessment was conducted in 2008. A total of 1474 persons (78.7%) were successfully interviewed in 2008. Those who could not be interviewed consisted of 112 (6.0%) who had died, 275 (14.7%) who had relocated or could not be found after repeated visits (a maximum of 5 visits were made) and 13 (0.7%) who refused to be interviewed. Of the 1474 persons who were successfully interviewed, 1302 provided complete information about hearing and the correlates examined in this report.

The interviews were done by 24 trained interviewers, all of whom had at least 12 years (high school) education. Many interviewers had previously done field surveys and had experience of face-to-face interviews. Interviewers undertook two weeks of training, consisting of an initial 6-day training given by one of the authors (OG) (which included item-by-item description of questionnaires and role play), followed by a further two days of debriefing and review after every interviewer had done two practice interviews in the field. Six supervisors, all of whom were university graduates and had survey experience, underwent the same level of training and monitored the day-to-day implementation of the survey.

### 2.3 Measures

Along with several other assessments, a checklist of chronic physical and pain conditions was included in the Ibadan Study of Ageing.^9^ At the 2008 follow-up respondents were asked if they had been told by a physician that they had diabetes or hypertension. The Rose Angina Questionnaire^10^ was used to assess presence of angina. Questions were asked about hearing-related problems. Specifically, respondents were asked: if they had (a) ‘difficulty hearing clearly’; (b) ‘recurrent pus discharge from the ear in the past’; (c) ‘recurrent nasal congestion and rhinorrhoea’ and (d) ‘any previous head injury’. Respondents were required to give a yes or no answer to each of these questions. The diagnoses of recurrent suppurative otitis media and recurrent rhinosinusitis were made based on positive response to questions ‘b’ and ‘c’ respectively. At the end of assessment, lasting an average of one hour, interviewers completed a set of questions reflecting their observation during the interview. One of the items was whether difficulty of hearing had been noted during the interview. In this report, only persons with reported hearing difficulty that was complemented by interviewer observation were regarded as having HI.

### 2.4 Data analysis

We present the unweighted estimates of the occurrence of HI. Univariate analysis was used to determine the significance of the differences in the occurrence of the demographic and clinical variables between the subjects with and without HI. Associations with socioeconomic variables and comorbid conditions were explored using logistic regression and the results are presented as odds ratios (ORs) with 95% confidence intervals. Economic status was assessed by taking an inventory of household and personal items such as chairs, clocks, buckets, radios, television sets, fans, stoves or cookers, cars and telephones; the list was composed of 21 such items. This is a standard and validated method of estimating economic wealth of elderly persons in low income settings.^7^ Respondents’ economic status is categorized by relating each respondent's total possessions to the median number of possessions of the entire sample. Thus, economic status is rated low if its ratio to the median is 0.5 or less, low-average if the ratio is 0.5–1.0, high-average if it is 1.0–2.0, and high if it is over 2.0. Residence was classified as rural (less than 12 000 households), semi-urban (12 000–20 000 households) or urban (greater than 20 000 households). The odds for the occurrence of variables were determined with multivariate analysis. The clinical correlates were explored with logistic regression analysis after adjusting for age^11^ and the estimates of standard errors of the ORs obtained were made with Stata version 7.0 (Stata Corp., College Station, TX, USA). All of the confidence intervals reported are at 95% and are adjusted for design effects. In order to take account of the sample design, we used the jackknife replication method implemented with the STATA statistical package to estimate standard errors for the means and proportions.^12^ Statistical significance was set at 0.05 in two-sided tests.

## 3 Results

The study sample consisted of 750 (57.6%) females and 552 (42.4%) males with a mean age of 77.3 years (SD = 0.3). Most respondents resided in rural or semi-urban households; 1002 (77.0%) and 704 (54.1%) had no formal education (Table 1). Seventy-nine respondents reported hearing loss which was confirmed by observers (Table 2), giving a prevalence of 6.1% (SD = 1.2). Residence was rural in 22 (27.8%) respondents, 41 (51.9%) smoked cigarettes and 40 (50.6%) drank alcohol.

**Table 1:** Demographic features of 1302 respondents participating in the Ibadan Study of Ageing

	*n* (%)
Age (years)	
65–69	189 (14.5)
70–74	363 (27.9)
75–79	267 (20.5)
≥80	483 (37.1)

Gender	
Female	750 (57.6)
Male	552 (42.4)

Education (years completed)	
≥13	97 (7.5)
7–12	173 (13.3)
1–6	328 (25.2)
0	704 (54.1)

Economic	
High	110 (8.5)
High-average	399 (30.6)
Low-average	488 (37.5)
Low	305 (23.4)

Residence	
Urban	300 (23.0)
Semi-urban	562 (43.2)
Rural	440 (33.8)

**Table 2:** Logistic regression analysis of sociodemographic and lifestyle correlates of hearing impairment (HI) in 79 study respondents

Variable	Unweighted number with HI	Odds ratio^a^	(95% CI)	*P*-value
Age (years)				
≥80	43	Reference		
75–79	19	0.8	(0.45–1.37)	NS
70–74	12	0.3	(0.18–0.67)	0.01
65–69	5	0.3	(0.11–0.71)	0.02

Gender				
Female	47	Reference		
Male	32	1.2	(0.75–1.89)	NS

Education (years completed)				
≥13	8	Reference		
7–12	10	0.7	(0.22–2.42)	NS
1–6	20	1.0	(0.36–2.85)	NS
0	41	1.0	(0.38–2.62)	NS

Economic status				
High	3	Reference		
High-average	24	2.1	(0.62–7.28)	NS
Low-average	29	1.9	(0.55–6.24)	NS
Low	23	2.4	(0.69–8.11)	NS

Residence				
Urban	17	Reference		
Semi-urban	40	1.2	(0.66–2.17)	NS
Rural	22	0.8	(0.42–1.55)	NS

Alcohol drinking				
No	39	Reference		
Yes	40	0.8	(0.52–1.33)	NS

Cigarette smoking				
No	38	Reference		
Yes	41	1.2	(0.78–1.97)	NS

NS: not significant

a Adjusted for sex, age or both, as appropriate

Univariate analysis comparing persons with HI and those without, revealed that age (*P* = 0.01) was significant while gender (*P* = 0.41), formal education (*P* = 0.86), economic status (*P* = 0.34), residence (*P* = 0.36), alcohol consumption (*P* = 0.42) and cigarette smoking (*P* = 0.17) were found to be non-significant.

Further logistic regression analysis in which gender or age was controlled for as appropriate, showed that age still remained a significant correlate with the likelihood of HI increasing with increasing age. In contrast, gender, formal education, economic status, residence, cigarette smoking or alcohol consumption were not significant correlates of HI (Table 2).

Various putative risk factors were examined for association with HI. These included previous recurrent suppurative otitis media, head injury, recurrent rhinosinusitis, angina, hypertension and diabetes mellitus. Of the 79 respondents with HI, 32 (40.5%) reported none of the risk factors. In a comparison between those who reported none of the assessed risk factors with those who reported one or more, 47 (59.5%) revealed no significant difference in the mean age, gender, educational grade, economic status or residence.

Table 3 shows the results of univariate analysis comparing the clinical correlates of elderly subjects with and without HI. History of previous recurrent suppurative otitis media (*P* = 0.01), head injury (*P* = 0.05) and current hypertension (*P* = 0.05) were found to be significant correlates while the other variables were not. However after adjusting for age and gender in a logistic regression analysis, self-reported history of recurrent suppurative otitis media (OR = 4.6, 95% CI 2.34–8.99, *P* = 0.01), head injury (OR = 2.2, 95% CI 1.14–4.26, *P* = 0.02) and hypertension (OR = 2.1, 95% CI 1.18–3.57, *P* = 0.01) were found to be significantly associated with HI (Table 4). On the other hand self-reported recurrent rhinosinusitis, transient ischemic attack and diabetes mellitus were not significant factors.

**Table 3:** Univariate analysis comparing clinical features in 1302 elderly subjects with and without hearing impairment

	Hearing impairment		*P*-value
	Yes	No	
	(*n* = 1223)	(*n* = 79)	
	*n* (%)	*n* (%)	
History of suppurative otitis media	257 (21.0)	4 (5.1)	0.01
History of head injury	134 (11.0)	4 (5.1)	0.05
Hypertension	110 (9.0)	4 (5.1)	0.05
History of recurrent rhinosinusitis	82 (6.7)	5 (6.3)	NS
Diabetes mellitus	92 (7.5)	5 (6.3)	NS
Transient ischemic attack	104 (8.5)	5 (6.3)	NS

NS: not significant.

**Table 4:** Logistic regression analysis of the clinical correlates of hearing impairment (HI) in 79 study respondents

	Unweighted number with HI	Odds ratio^a^	(95% CI)	*P*-value
History of suppurative otitis media	13	4.6	(2.34–8.99)	0.01
History of head injury	12	2.2	(1.14–4.26)	0.02
History of recurrent rhinosinusitis	9	1.1	(0.52–2.20)	NS
Transient ischemic attack	6	1.4	(0.60–3.44)	NS
Diabetes mellitus	4	1.5	(0.51–3.57)	NS
Hypertension	19	2.1	(1.18–3.57)	0.01

NS: not significant

a adjusted for age.

## 4 Discussion

In this study we have estimated a prevalence of 6.1% for hearing loss in elderly Nigerians aged ≥65 years. We have shown that the prevalence of HI significantly increased with age but had no relationship with other socioeconomic and lifestyle factors. In addition, HI was significantly related to self-reported suppurative otitis media and head injury as well as assessed hypertension.

The estimated prevalence of HI in this sample is close to that reported by Bazargan et al.^5^ who reported fair to poor hearing in between 9.3% and 26% of their sample. However, our estimate was low when compared with the 30% prevalence of HI reported in 977 elderly people residing in a community in the UK^13^ and 21% of those aged ≥60 in the Alameda County Study in California, USA.^1^ Our observation of age as a significant risk factor agrees with the assertion made by Moscicki et al.^14^ and others^1^, ^5^, ^13^ that advanced age might be the most important risk factor for hearing loss. One factor which might predispose to higher occurrence of HI in the elderly is the exposure to multiple medications, common in the very old. Multiple medications have been reported to produce a cumulative deleterious effect on hearing.^15^, ^16^, ^17^ That is, when only one drug was administered, it might not be ototoxic, but in conjunction with other medications, or noise, it could affect an individual's hearing. The myriad number of medications which could be obtained across the counter in our study setting and the nearly unlimited number of possible combinations in which they could be used, makes this hypothesis very difficult to explore in our study.

Even though a higher estimate of HI was found in women compared with men, gender was not found to be significant in the occurrence of HI. There has been inconsistency in the report of gender as a risk factor for HI in the literature. For example, while some have found females to be at elevated risk,^4^, ^5^ others have not found this to be the case.^1^, ^3^, ^13^ These differences might reflect the differential exposure to putative risk factors between the genders in various study populations. In a previous report, while a history of noise exposure was found to be a significant risk factor for men, history of Meniere's disease and medical illness were found to be significant risk factors for women.^18^, ^19^, ^20^, ^21^, ^22^ Furthermore, access to health care might also be important in determining the rate of HI between men and women. Generally in Africa, the economic status of women tends to be lower than that of men and this may reduce access to healthcare.

The main risk factors identified in this report were previous suppurative otitis media, head injury and hypertension. Macandie and O’Reilly^22^ reported that the differences in preoperative HI (assessed using bone conduction threshold) between the ears with previous suppurative otitis media and contra-lateral normal ears were statistically significant (*P* < 0.01). Similarly, Cusimano et al.^19^ reported that the sensorineural component in hearing loss correlated with the duration of chronic otitis media. All of these previous observations would appear to support the significance of previous suppurative otitis media in the development of HI as found in our study. In another study, post-traumatic HI was reported in 60% of patients with head injury; 43% was conductive while 52% was sensorineural.^21^ Evidence showed that while concussion injury of the inner ear structure was the principal mechanism of post-traumatic HI, ossicular injuries, labyrinthine contusion and petechial hemorrhages into the brainstem have also been implicated.^23^

Based on the proportion of elderly with HI who were negative for any of the assessed risk factors in this study, it could be assumed that presbyacusis probably accounted for 41% of the HI in the elderly. This proportion was high compared to 16% reported by Mościcki et al.^14^ Those authors interpreted their findings to mean that an unexplained hearing loss in an elderly person can, in fact, be explained in most instances by age. However, this high frequency of presbyacusis would not rule out the possibility that there might be other etiological factors of HI in a large proportion of the elderly, which were not considered in our study.

Gilad and Glorigl^18^ regarded hearing loss among the elderly as the culmination of multiple, damaging processes throughout an individual's life; other investigators have suggested cardiovascular risk as the major factors.^5^, ^13^ This might explain the significance of hypertension as found in this study. Also, some other workers have suggested a role for genetics and immunobiology in the occurrence of HI in the elderly.^18^, ^20^ Cumulatively, it would appear that middle-aged and older people with a genetic vulnerability to hearing loss should be particularly careful about environmental risk factors such as harmful noises and medications whose side effects could be detrimental to hearing.^20^ One important limitation of this study was that acoustic trauma due to noise, which is becoming a significant health problem in many societies, was not addressed. The use of a self-report questionnaire survey to gather information would have made the assessment of relative noise exposure open to significant recall bias. We found a non-significant trend for HI to be less prevalent among rural compared to urban elderly residents. This trend might reflect the observation that hearing levels were poorer in urbanized communities^23^ than in isolated or agrarian societies,^24^ as is the case in Nigeria and other sub-Saharan African countries.

In agreement with three previous reports^24^, ^25^, ^26^ our study has not established a positive association of HI with smoking and alcohol drinking, a finding that was in contrast to two others^27^, ^28^ whose surveys found smoking to be a significant risk factor. The suggestion has been made that cigarette smoking increases the body's need for oxygen because the carbon monoxide that is inhaled with smoke could partially block hemoglobin.^27^ In addition smoking was implicated in the reduction of blood supply to the cochlear because of vasospasm caused by nicotine as well as atherosclerotic narrowing of vessels and thrombotic occlusion by aggregation of platelets.^27^, ^29^ This has led to the speculation that smoking might adversely affect the vascular system of the inner ear. In addition, histopathological studies of ageing gerbils have provided strong evidence for vascular involvement in age-related hearing loss.^29^ Morphometric analyses of lateral wall preparations stained to contrast blood vessels have shown losses of strial capillary area in aged animals. The vascular pathological changes first occurred as small focal lesions mainly in the apical and lower basal turns and progressed with age to encompass large regions at both ends of the cochlea.^29^, ^30^

In conclusion, this report has documented the prevalence of HI in the elderly in Nigeria which is comparable to those reported for other elderly populations. It also identified age, and controllable factors such as previous suppurative otitis media, head injury and hypertension, as correlates for HI. However, the high proportion of presumed presbyacusis suggests a need for further study in this group. The implication of this study is that the control of preventable or ameliorable factors and provision of hearing aids should be an important item in any health care policy initiative for elderly persons in the other countries in the sub-Saharan African subregion which also share similar epidemiological parameters to Nigeria. At the present time, most elderly persons in need are unable to afford this important device that may help improve their quality of life.

## Authors’ contributions

OG conceived and designed the study; OG, AOL and TA analysed and interpreted the data; AOL drafted the article; OG and AOL revised the article critically for intellectual content. All authors read and approved the final manuscript. OG is guarantor of the paper.

## Funding

The Wellcome Trust, London, UK provided funding for the Ibadan Study of Ageing (grant no. WT079662MF). The Wellcome Trust was not involved in the collection, analysis and interpretation of the data.

## Conflicts of interest

None declared.

## Ethical approval

University of Ibadan/University College Hospital, Ibadan Joint Ethical Review Board, Ibadan, Oyo State, Nigeria.
